# State-of-the-Art and Practical Guide to Ultrasonic Transducers for Harsh Environments Including Temperatures above 2120 °F (1000 °C) and Neutron Flux above 10^13^ n/cm^2^

**DOI:** 10.3390/s19214755

**Published:** 2019-11-01

**Authors:** Bernhard R. Tittmann, Caio F.G. Batista, Yamankumar P. Trivedi, Clifford J. Lissenden, Brian T. Reinhardt

**Affiliations:** 1 Department of Engineering Science and Mechanics, Penn State University, University Park, PA 16802, USA; YPT5002@psu.edu (Y.P.T.);; 2 Olympus Corp., State College, PA 16801, USA; caio.batista@outlook.com; 3 Applied Research Laboratory, Penn State University, University Park, PA 16802, USA; nairbt85@gmail.com

**Keywords:** piezoelectric, high-temperature ultrasonic testing, radiation resistance, field-deployable sensor, guided-wave send–receive, spray-on transducers, piezocomposites

## Abstract

In field applications currently used for health monitoring and nondestructive testing, ultrasonic transducers primarily employ PZT5-H as the piezoelectric element for ultrasound transmission and detection. This material has a Curie–Weiss temperature that limits its use to about 210 °C. Some industrial applications require much higher temperatures, i.e., 1000–1200 °C and possible nuclear radiation up to 10^20^ n/cm^2^ when performance is required in a reactor environment. The goal of this paper is the survey and review of piezoelectric elements for use in harsh environments for the ultimate purpose for structural health monitoring (SHM), non-destructive evaluation (NDE) and material characterization (NDMC). The survey comprises the following categories: 1. High-temperature applications with single crystals, thick-film ceramics, and composite ceramics, 2. Radiation-tolerant materials, and 3. Spray-on transducers for harsh-environment applications. In each category the known characteristics are listed, and examples are given of performance in harsh environments. Highlighting some examples, the performance of single-crystal lithium niobate wafers is demonstrated up to 1100 °C. The wafers with the C-direction normal to the wafer plane were mounted on steel cylinders with high-temperature Sauereisen and silver paste wire mountings and tested in air. In another example, the practical use in harsh radiation environments aluminum nitride (AlN) was found to be a good candidate operating well in two different nuclear reactors. The radiation hardness of AlN was evident from the unaltered piezoelectric coefficient after a fast and thermal neutron exposure in a nuclear reactor core (thermal flux = 2.12 × 10^13^ ncm^−2^; fast flux 2 (>1.0 MeV) = 4.05 × 10^13^ ncm^−2^; gamma dose rate: 1 × 10^9^ r/h; temperature: 400–500 °C). Additionally, some of the high-temperature transducers are shown to be capable of mounting without requiring coupling material. Pulse-echo signal amplitudes (peak-to-peak) for the first two reflections as a function of the temperature for lithium niobate thick-film, spray-on transducers were observed to temperatures of about 900 °C. Guided-wave send-and-receive operation in the 2–4 MHz range was demonstrated on 2–3 mm thick Aluminum (6061) structures for possible field deployable applications where standard ultrasonic coupling media do not survive because of the harsh environment. This approach would benefit steam generators and steam pipes where temperatures are above 210 °C. In summary, there are several promising approaches to ultrasonic transducers for harsh environments and this paper presents a survey based on literature searches and in-house laboratory observations.

## 1. Introduction

Currently, ultrasonic non-destructive evaluation (NDE) is employed periodically on passive high temperature components, but continuous online monitoring has not been widely implemented. The need for continuous online monitoring is becoming more important with the need for high-temperature infrastructure license extension. Additionally, ultrasound is a highly attractive NDE methodology given that it allows for inspection in optically opaque materials, such as liquid-metal coolants, steam generator piping, and heat exchanger pipes. Further applications may be found in materials research reactors where ultrasonic NDE can be used for in situ analysis of radiation effects on novel radiation-hard materials currently being developed.

During the past decades there has been significant interest and therefore research into the problem of ultrasonic transducers for harsh environments [1,2,3,4,5,6,7,8,9,10,11,12,13,14,15,16,17,18,19,20,21,22,23,24,25,26,27,28,29,30,31,32,33,34,35,36,37,38,39,40,41,42,43,44,45,46,47,48,49,50,51,52,53,54,55,56,57,58,59,60,61,62,63,64,65,66,67,68,69,70,71,72,73,74,75,76,77,78,79,80,81,82]. The aim of this paper is to give an overview, review, and survey of piezoelectric materials for possible harsh-environment applications. The survey is conveniently divided into several categories: single crystals, piezoelectric ceramics, composite ceramics, and radiation-resistant materials and the new category of brush-on transducers. The survey starts with several relatively well-known high-temperature piezoelectric materials summarized in Table 1 for comparison. Listed also are the Curie–Weiss temperatures, which are useful in that they provide a limit to the temperature to which a material can exhibit piezoelectricity. Furthermore, the conventional PZT 5H is also listed, which is the commonly used piezoelectric in commercial applications.

## 2. Transducers for High Temperature Applications

### 2.1. Single-Crystal Wafers

**Figure 1:**
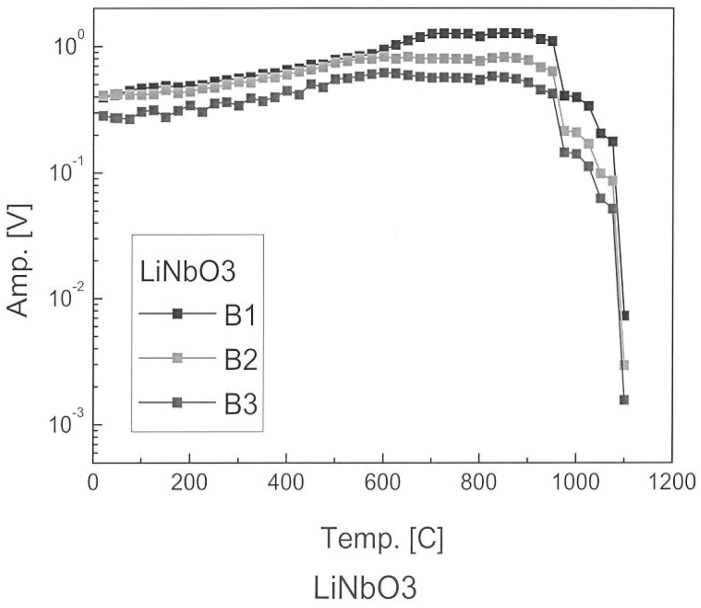
Temperature profiles for pulse-echo amplitude of lithium niobate (LiNbO_3_) single crystal bonded to steel. B1, B2, and B3 describe three successive runs [1].

**Figure 2:**
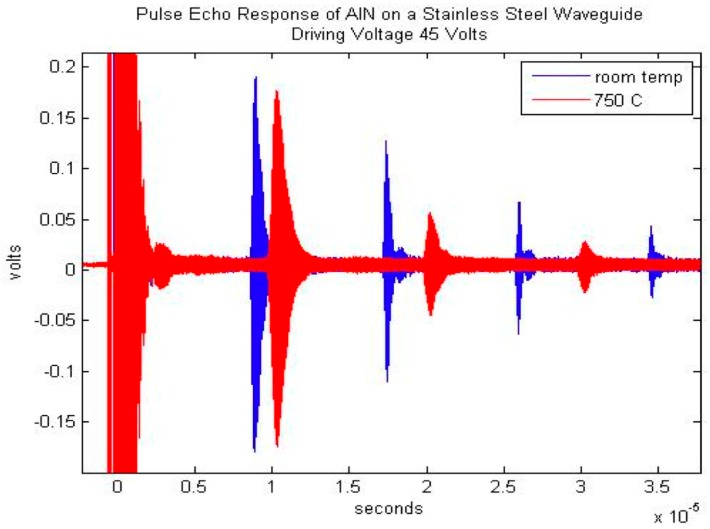
Pulse-echo amplitude response for a single-crystal wafer of aluminum nitride at two temperatures, 25 and 750 °C, showing only somewhat lower amplitudes at the higher temperature [2].

**Figure 3:**
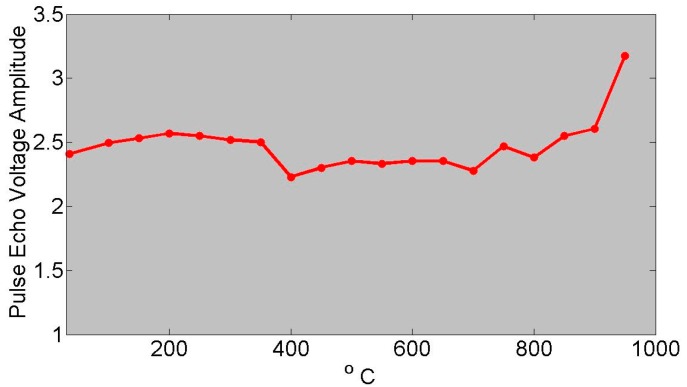
Ultrasonic high-temperature performance of single-crystal AlN wafer on steel cylinder showing acceptable performance to about 950 °C [2].

In the category of the single crystals, both maximum temperature and long-term in situ operation were investigated in a comparison study. These is the well-known lithium niobate (LiNbO_3_), and then two relatively recently developed materials [3]: aluminum nitride (AlN) and YCOB [YCa_4_O(BO_3_)_3_]. As shown in Figure 1, the pulse-echo amplitude of LiNbO_3_ is stable until about 1000 °C [1]. Figure 2 shows the pulse-echo amplitude response for a single-crystal wafer of aluminum nitride at two temperatures, 25 and 750 °C, showing only somewhat lower amplitudes at the higher temperature [2]. Figure 3 shows the ultrasonic high-temperature performance of single-crystal AlN wafer coupled to a steel cylinder with acceptable performance to about 950 °C [2].

**Figure 4:**
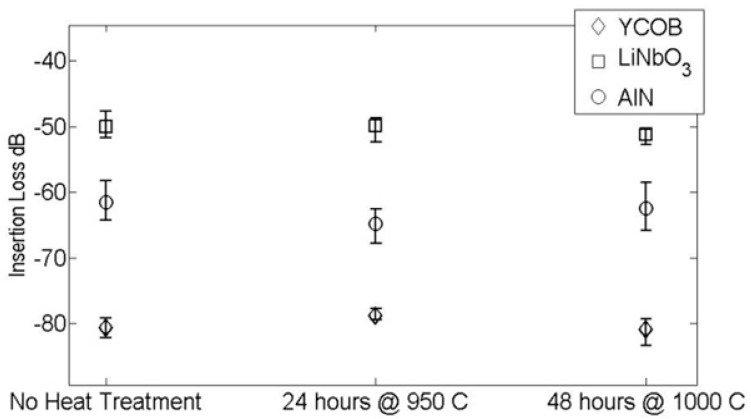
Comparison of heat treatment results for lithium niobate (LiNbO_3_), aluminum nitride (AlN), and YCOB [YCa_4_O(BO_3_)_3_] [3].

Shown in Figure 4 are three consecutive runs over a measurement time of 14 h [3]. As can be seen, all three materials exhibited stability in ultrasonic performance through heat treatment of 950 °C for 24 h and 1000 °C for 48 h. This “cook-and-look” testing revealed significant changes in the dielectric properties and only small changes in the ultrasonic performance of lithium niobate. Dielectric changes of the observed magnitude would be expected to have a noticeable effect on the ultrasonic performance. However, the heat treatments were not equivalent during the dielectric and ultrasonic testing. It is quite likely that the longer heat treatment caused a more pronounced change in the dielectric properties of the lithium niobate [3].

The YCOB on the other hand exhibited a much less pronounced change in dielectric properties after heat treatment. It is expected that YCOB is more stable at high temperatures than LiNbO_3_ which is known to deplete its oxygen particularly at low oxygen partial pressure [3].

**Figure 5:**
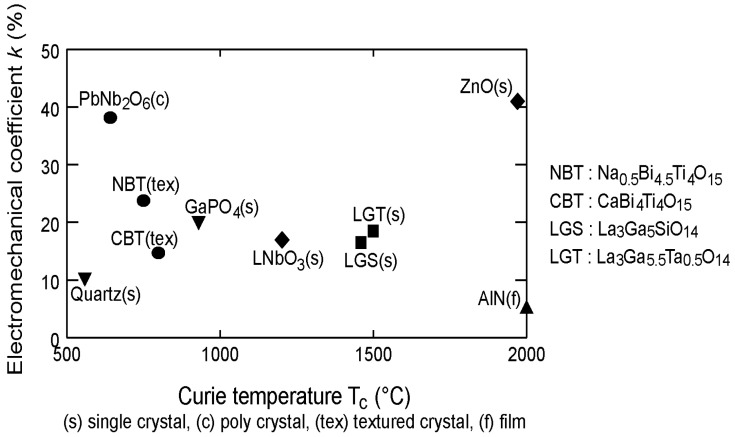
Electromechanical coefficient versus Curie temperature T*_c_* [10].

Material selection is based primarily on combining Curie temperatures (T*_c_*) and coupling coefficients (e.g., d_33_) of the constituents to achieve the desired overall piezoceramic properties. To maintain an in-field transducer at high signal-to-noise (SNR), the piezoelectric transducer material should have both a large coupling coefficient and a T*_c_* exceeding the transducer’s operating temperature [4,5,6,7,8,9,10]. Micromechanical modeling enables prediction of overall properties based on the properties of the constituents. Figure 5 presents a graph showing the electromechanical coefficient, d_33_, as a function of the T*_c_* for a selection of piezoelectric materials, consisting of single crystals, polycrystals, textured crystals, and films [10].

### 2.2. Thick-Film Ceramics

In the category of thick-film ceramic sample preparation, poling, acoustic data, high-temperature tests, and the effect of protective aluminum oxide layer on both poling and temperature performance were studied. Bismuth titanate thick-film transducers performed well up to 600 °C. Currently, tests are ongoing with thick-film transducers deposited on pipes and simulated casings for NDE with guided waves generated by both flat and curved arrays. Recently developed piezoelectric materials with high Curie temperatures are listed in Table 2.

Conventional piston-type transducers that send and receive ultrasonic waves typically use lead zirconate titanate for the active element and have backing and matching layers. In addition, they are usually coupled to the substrate through gel or adhesive. Harsh environments limit the types of couplants that can be used, and curved surfaces present additional challenges. In contrast, spray-on transducers are bonded directly to the substrate, precluding the need for couplants. Spraying transducers onto curved surfaces is not substantially different from doing so on flat surfaces. No matching or backing layers are used in this work, but they could be used if deemed necessary. One advantage that spray-on transducers provide is the ability to design the transducer material for a specific operating temperature by mixing powders into a sol gel to create a composite (or alloy). 

### 2.3. Composite Ceramics

**Figure 6:**
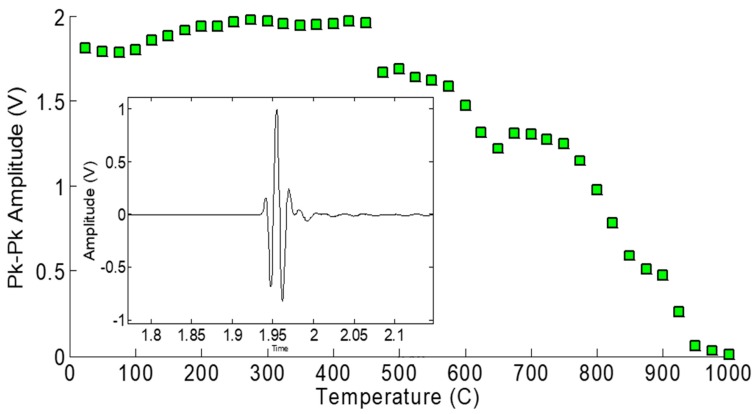
Temperature dependence of pulse-echo amplitude for PZT/Bi_4_Ti_3_O_12_ piezocomposite spray-on transducers deposited on steel cylinders [29].

**Figure 7:**
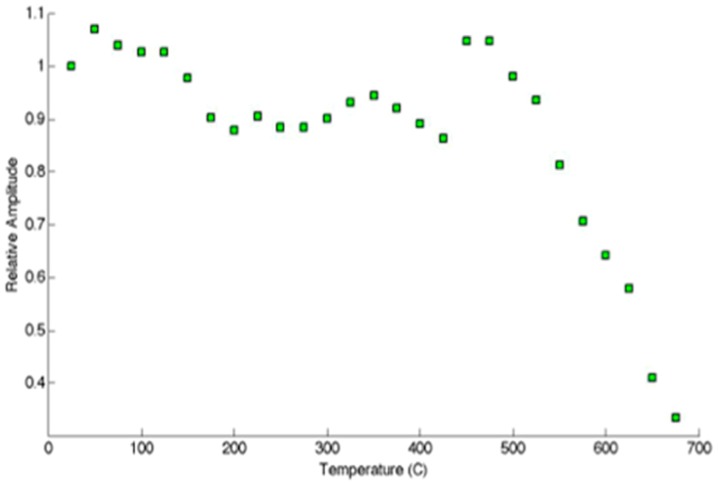
Temperature dependence of pulse-echo amplitude for Bi_4_Ti_3_O_12_/LiNbO_3_ piezocomposite spray-on transducer [29].

The biggest difference between piezoelectric materials used in conventional transducers and spray-on piezoelectric transducers is density/porosity. Pressure is an integral part of forming fully dense piezoceramics, and it is not part of spray-on processing. Thus, spray-on transducers have porosity that affects their properties. On the positive side, it also provides strain tolerance to the piezoceramic, which is bonded to a metal substrate that is subject to temperature changes. The pioneers of spray-on piezoelectric transducer technology are Barrow and Kobayashi. Barrow et al. [18,19] added powder to sol gel to form piezoelectric films thicker than 1–2 μm using a spin coating methodology. Kobayashi et al. [20,21,22,23,24] then adapted the powder/sol–gel technique using a spray gun to deposit films on metal substrates. Searfass et al. [25,26,27,28] have provided technological advancements on the sol–gel composition, fabrication, characterization, and high-temperature ultrasonic testing for such spray-on transducers. As examples, Figure 6 and Figure 7 show that PZT/Bi_4_Ti_3_O_12_ and Bi_4_Ti_3_O_12_/LiNbO_3_ composite transducers mounted on steel cylinders functioned well in pulse-echo mode until 675 and 1000 °C, respectively.

The PZT/Bi_4_Ti_3_O_12_ transducer’s efficiency decreased when operating in pulse-echo mode, but a discernable signal was still observed as low as 500 kHz. The thickness of this transducer was still relatively thin, especially for low-frequency operation. The broadband nature of this transducer was very evident in its testing in that it had a center frequency around 2.75 MHz but could still operate effectively well below 1 MHz.

The Bi_4_Ti_3_O_12_/LiNbO_3_ transducer was also tested for low-frequency operation, but it was considerably less efficient. The signal effectively disappeared at frequencies much below 1 MHz. This again shows the great advantage of the use of the PZT/Bi_4_Ti_3_O_12_ composite. The PZT/Bi_4_Ti_3_O_12_ has a much greater signal amplitude and is more broadband allowing it to operate at low frequencies and produce viable waveforms. Thicker PZT/Bi_4_Ti_3_O_12_ transducers may further enhance their operation at low frequencies. Both the signal amplitude and signal-to-noise ratio can be increased along with better operation in the pulse-echo mode [25,26,27,28].

## 3. Piezoelectric Materials for Radiation Environment

Ultrasonic measurements have a long and successful history of use for material characterization, including detection and characterization of degradation and damage, measurement of various physical parameters used for process control, such as temperature and fluid flow rate, and in nondestructive evaluation (NDE) [31]. However, application of ultrasonic sensors in nuclear reactors has been limited to low neutron flux environments. The development of ultrasonic tools to perform different in-pile measurements requires a fundamental understanding of the behavior of ultrasonic-transducer materials in these high neutron flux environments. Irradiation studies of ultrasonic transducers have been described in the literature but are generally at lower flux/fluences than what might be seen in U.S. nuclear reactors. The Pennsylvania State University (PSU) lead an effort that was selected by the Advanced Test Reactor National Scientific User Facility (ATR-NSUF) for an irradiation of ultrasonic transducers in the Massachusetts Institute of Technology Nuclear Research Reactor (MITR) [32,33]. This test was an instrumented-lead test, allowing real-time signals to be received from five ultrasonic transducers including three piezoelectric transducers two of which were single-crystal wafers of aluminum nitride. The irradiation began on February 20th, 2014 and was scheduled to run for a period of 18 months or until all the sensors have ceased to operate. Recent results are presented and discussed in detail in References [32,33]. In searching for candidate materials for use in harsh environments, the most straightforward down selection parameter seems to be the transition temperature, which provides an upper limit on the operating range of the piezoelectric material. In fact, a higher Curie temperature has been found to correlate with increased radiation tolerance and the primary effect of radiation damage in piezoelectric materials appears to be depolarization [21]. A table of candidate materials for longitudinal wave generation is provided below in Table 3; however, this is only the first step. The final column in Table 3 is of substantial importance as it has been found that crystal structure plays a significant role in radiation tolerance of ceramics [33,34,35,36,37,38,39,40,41,42,43,44,45,46,47,48,49,50,51,52,53,54,55,56,57,58,59,60,61,62,63,64,65,66,67,68,69,70,71,72,73,74].

For the radiation effects, the discussion focuses on the case of AlN, which is not a ferroelectric and has a transition temperature of 2865 °C (melting temperature). We also consider the case in which the bulk of the crystal is kept below any transition temperature. In this scenario, during irradiation four primary forms of damage are anticipated in a piezoelectric material:

(1) depoling via thermal spike processes,
(2) morphization/metamictization due to displacement spikes or high concentration of point defects,
(3) increase in point defect concentration, and
(4) development of defect aggregates.

Here, only the two most likely damage mechanisms are summarized, namely thermal spikes and displacement spikes [33]. Additionally, transmutation products are considered, as these in fact induce both thermal spikes and displacement spikes in some cases. To summarize, the considerations lead to the conclusion that AlN is resistant to amorphization. Moreover, the very high transition temperature renders the material immune to thermal spike damage. It is also clear that the transmutation reaction, ^14^N(n,p)^14^C, generates only a fraction of a dpa at 10^21^ n/cm^2^ and insignificant doping.

**Figure 8:**
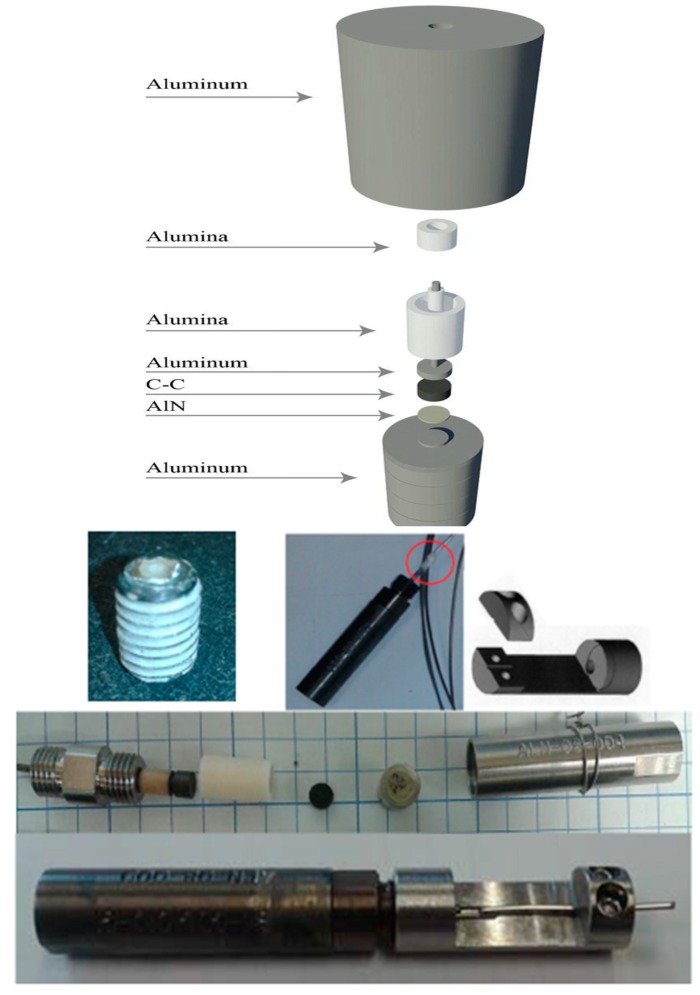
Photos of the fixture inserted into the Massachusetts Institute of Technology (MIT) reactor [31].

A single-crystal AlN element (4.8 mm in diameter and 0.45 mm thick) resonant at 13.4 MHz, was coupled to an aluminum cylinder via mechanical pressure. Aluminum foil was used as an acoustic coupler between the aluminum cylinder and the AlN element, allowing for strong, clear A-scan data to be obtained. The AlN element was loaded, on the side opposite the aluminum cylinder, with a sintered carbon/carbon composite to reduce ringing and improve the signal clarity. The test fixture is illustrated in Figure 8.

The aluminum cylinder acted as the lower electrical contact and the plunger provided the upper electrical contact. The setup was connected to a radiation-hard 50 ohm coaxial cable. This radiation-hard cable consisted of an aluminum conduit sleeve over fused quartz dielectric tubing with an aluminum inner conductor. The cylinder/piezo setup was placed in the core of the Penn State TRIGA reactor and irradiated to a fast and thermal neutron fluence of 1.85 × 10^18^ n/cm^2^ and 5.8 × 10^18^ n/cm^2^, respectively, and a gamma dose of 26.8 MGy. Throughout the irradiation the A-scan data were recorded with impedance measurements interspersed. 

A similar fixture was built and inserted into the reactor at the Massachusetts Institute of Technology (MITR) for the ATR-NSUF tests. Table 4 gives the MIT Research Reactor Environment. Figure 8 shows a photo of the fixture before being inserted into the MITR.

### 3.1. Temperature Tolerance

**Figure 9:**
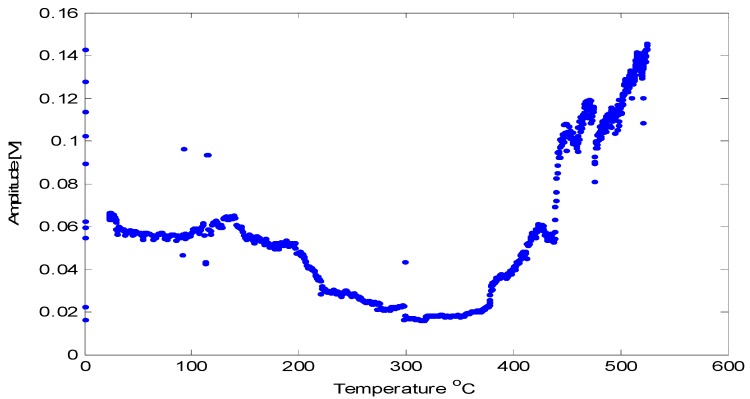
Relative pulse-echo amplitude measured as function of temperature for AlN sample. Note the increase as temperature is raised above 400 °C [31].

**Figure 10:**
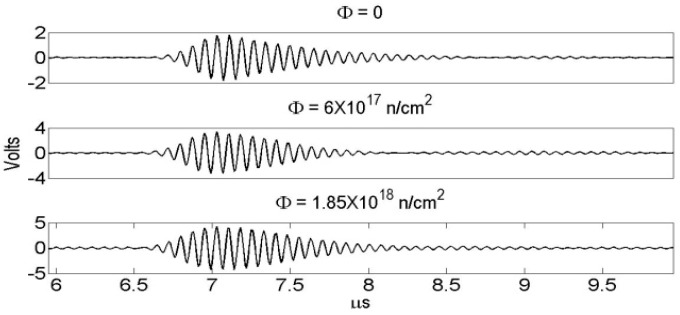
A-Scans obtained from AlN TRIGA reactor core, Φ is the fast neutron fluence [31].

Prior high-temperature experiments with AlN [2,3] may lead one to suspect that crystalline defects can degrade the high-temperature transduction of AlN. Considering that radiation causes displacement damage and transmutation doping, one may wonder how the irradiated AlN would fare at high temperatures. To answer this call the irradiated crystal, having negligible activity after cooling for a few weeks, was tested up to 500 °C. Figure 9 shows the relative pulse-echo amplitude measured as a function of temperature. Some of the waveforms are provided in Figure 10. Additionally, d_33_ was measured prior to and after irradiation and found to be 5.5 pC/N, which is unchanged from the pristine value. Further, subjecting the irradiated AlN crystal to temperatures of 950 °C for 72 h caused no change in the performance of the AlN crystal [32,33,34].

### 3.2. Radiation Tolerance

**Figure 11:**
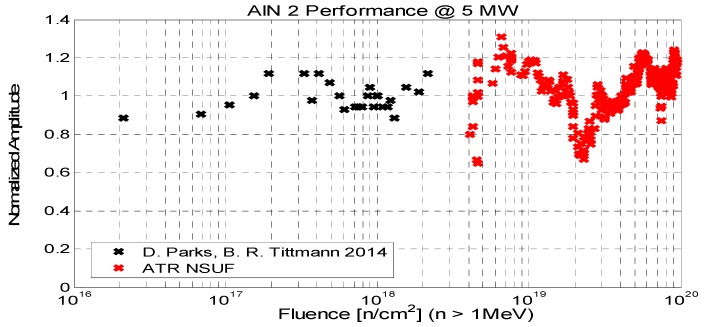
Normalized amplitude of pulse-echo signal showing the results of both the Pennsylvania State University (PSU) TRIGA reactor and the MITR (Massachusetts Institute of Technology Nuclear Research Reactor) measurements. Note that the excursions to low amplitudes are the results of reactor scrams [31].

The A-scan data, illustrated in Figure 10, were recorded and analyzed in terms of the echo amplitude, which are presented in Figure 11. The amplitude over the course of irradiation remains nearly constant and indicates the radiation hardness of the AlN and the test fixture. In Figure 11 the black dots represent the data from the tests in the Penn State TRIGA reactor, whereas the red points represent the data from the tests in the MITR.

For practical use in harsh radiation environments, the selection criteria for piezoelectric materials for NDE and material characterization were summarized. Using these criteria piezoelectric aluminum nitride was shown to be a viable candidate. The results of tests on an aluminum-nitride-based transducer operating in two nuclear reactors were presented. The tolerance of single-crystal piezoelectric aluminum nitride after fluences of up to 10^20^ n/cm^2^ is examined. The radiation hardness of AlN is most evident from the unaltered piezoelectric coefficient d_33_, which measured 5.5 pC/N after a fast and thermal neutron exposure in a nuclear reactor core for over 120 MWh in agreement with the published literature value. The results offer potential for improving reactor safety and furthering the understanding of radiation effects on materials by enabling structural health monitoring and NDE in spite of the high levels of radiation and high temperatures known to destroy typical commercial ultrasonic transducers.

## 4. Spray-On Transducers for Harsh Environment Applications

Damage detection in the power industry is always vying for optimized and cheaper techniques. Most components in the energy sector utilize metallic structure, whether it is for power generation, storage, transportation, or waste management. Many components operate at a high temperature adding further challenges for their health monitoring. Given that commercial transducers rated for elevated temperatures are limited and expensive, the use of spray-on film transducers for such purposes has been researched while keeping the fabrication simple enough for anyone to create them. 

Bismuth titanate (Bi_4_Ti_3_O_12_) is an excellent piezoelectric which has a T*_c_* of 670 °C and a safe operating level until about 500 °C, considerably higher than PZT. Unlike the preceding sol–gel method [18,19,20,21,22,23,24,25,26], this fabrication process involves a lithium-silicate-based inorganic binder and water to mix with the Bi_4_Ti_3_O_12_ powder. The following steps are optimized for best results.

**Figure 12:**
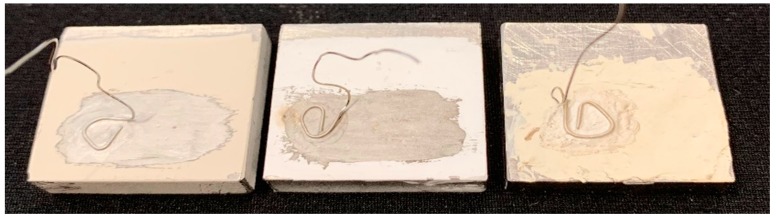
Bismuth titanate, lithium niobate, and organic bismuth titanate film transducers (left to right). Good adhesion was observed for extended periods of time. One film transducer is still working normally after several years of use [7].

1. Select the powder (BIT or lithium niobate/barium titanate) and mix with Ceramabind 830 to achieve a 1:0.2:0.8 ratio (powder–binder–water by weight ratio); A plastic stirrer was used to rigorously mix the powder and binder, but it could be mixed with a ultrasonic horn.
2. Create the solution by combining the mixed powder/binder with distilled water at the specified concentration in a 15 mL glass vial.
3. Prepare the substrate by roughening the surface with a fine-grit sandpaper, and then clean it with isopropyl alcohol.
4. Spray the slurry onto the substrate with an air gun (Goplus Electric Paint Sprayer, 450W High Power HVLP Paint Spray Gun with 3 Spray Patterns, 3 Nozzle Sizes, Adjustable Valve Knob and 900ml Large Detachable Container); The air gun pressure should be 20–22 psi and the nozzle should be approximately 20 cm from the surface. Alternatively, apply slurry with a brush.
5. Dry each layer of the sprayed film in the relatively low-humidity environment (15–20%) of a glove box for at least 15 min to avoid cracking.
6. Repeat steps 4 and 5 to achieve the desired film thickness (preferably thicker than 120 μm). The average thickness of a single spray is 18 μm.
7. Use a thickness gage to measure the average thickness of the film.
8. After the film layers have cured, brush apply a conductive silver paint (SPI Chemicals, Inc., Atlanta, GA, USA) on the portion of the film to become the transducer to a thickness of approximately 30 μm. Each layer takes approximately 15 min to cure in the low-humidity setting, so if there are eight spray repetitions, it will take about 2 h. For films thicker than eight layers, the cure time for a layer may be longer.
9. Once the electrode is applied, heat the sample to 60 °C for a few minutes with a heat gun to allow the electrode to dry. This step is optional as the electrode can air dry in a longer time.
10. Attach a bare nickel chrome wire (supplied by Consolidated) with silver paint to serve as the lead wire as shown in Figure 12.
11. Pole sample at a desired electric field for at least 20 min at ambient temperature.

With the initial bulk-wave characterization of the film, it was noted that despite the lower piezoelectric coefficient compared to PZT, the film transducers were able to function at higher temperatures. Another major advantage is the straightforward fabrication procedure and the ability for these films to cure at room temperature. An alternative method to produce these films consisted of using organic compounds instead of the high-temperature inorganic binder. The organic films were also excellent in heat resistance despite having a slightly complicated fabrication procedure compared to the inorganic films. Figure 12 shows samples of Bi_4_Ti_3_O_12_ (left), LiNbO_3_ (center), and organic Bi_4_Ti_3_O_12_ (right) spray-on films were fabricated using the beforementioned procedure and the inorganic method.

**Figure 13:**
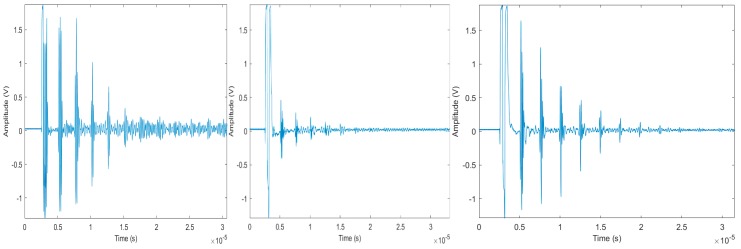
A-scan pulse-echo measurements of Bi_4_Ti_3_O_12_ (**left**), LiNbO_3_ (**center**), and organic Bi_4_Ti_3_O_12_ (**right**) thick-film transducers deposited on steel cylinder (7 mm) at 40 dB gain, film thickness ~200 micron, and frequency ~1.5 MHz [7].

Figure 13 shows A-scan pulse-echo measurements of Bi_4_Ti_3_O_12_ (left), LiNbO_3_ (center), and organic Bi_4_Ti_3_O_12_ (right) thick-film transducers deposited on a steel cylinder (7 mm) at 40 dB gain, film thickness ~200 micron, and frequency ~1.5 MHz [7].

The bismuth titanate powder used was 99.99% pure Bi_4_Ti_3_O_12_ 200 mesh (75 μm particle size) supplied by Lorad Chemical Corporation. Lithium niobate powder was a 99.99% pure LiNbO_3_ 325 mesh (45 μm particle size) supplied by LTS Research Laboratories, Inc. (Orangeburg, NY 10962, USA). The barium titanate used was 99% pure BaTiO_3_ 325 mesh (45 μm particle size) supplied by Acros organics (Thermo Fisher Scientific, Branchburg, NJ, USA).

**Figure 14:**
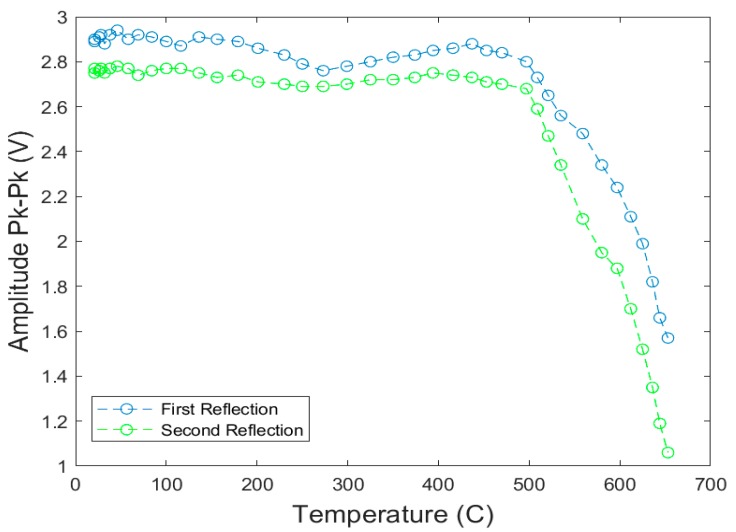
Signal amplitude (peak-to-peak) for the first two reflections as a function of temperature for bismuth titanate thick-film, spray-on transducer [7].

**Figure 15:**
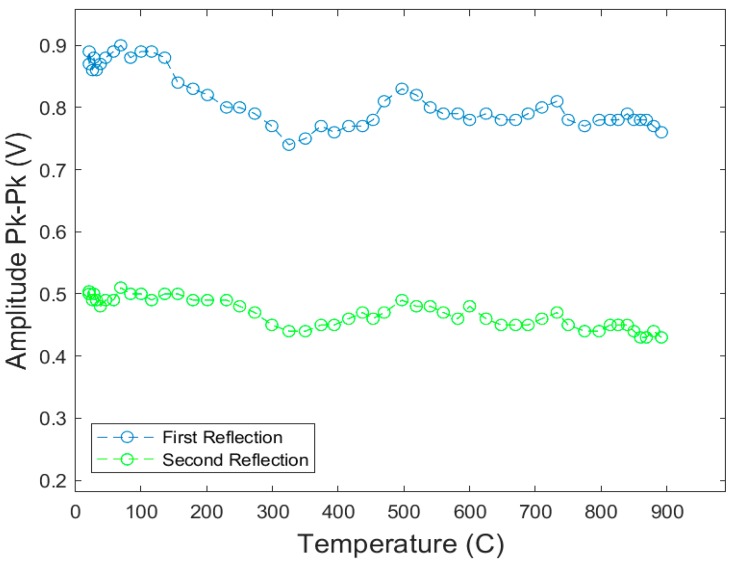
Signal amplitude (peak-to-peak) for the first two reflection as a function of the temperature for lithium niobate thick-film, spray-on transducer [7].

The Curie–Weiss temperatures of Bi_4_Ti_3_O_12_ and LiNbO_3_ make them ideal candidates for high-temperature testing. These films were inserted in the tube furnace and peak-to-peak voltage measurements for the first and second reflection from an edge were recorded. The furnace was set to increase the temperature of the films at a rate of about 6 °C/min. The Bi_4_Ti_3_O_12_ films were tested up to a temperature of 650 °C, whereas the LiNbO_3_ films were tested to a temperature of 900 °C. The first and second echoes were recorded in terms of the signal amplitude and plotted in relation with the temperature ramp seen here in Figure 14 for the Bi_4_Ti_3_O_12_ film. Figure 15 shows the results for LiNbO_3_ sample.

**Figure 16:**
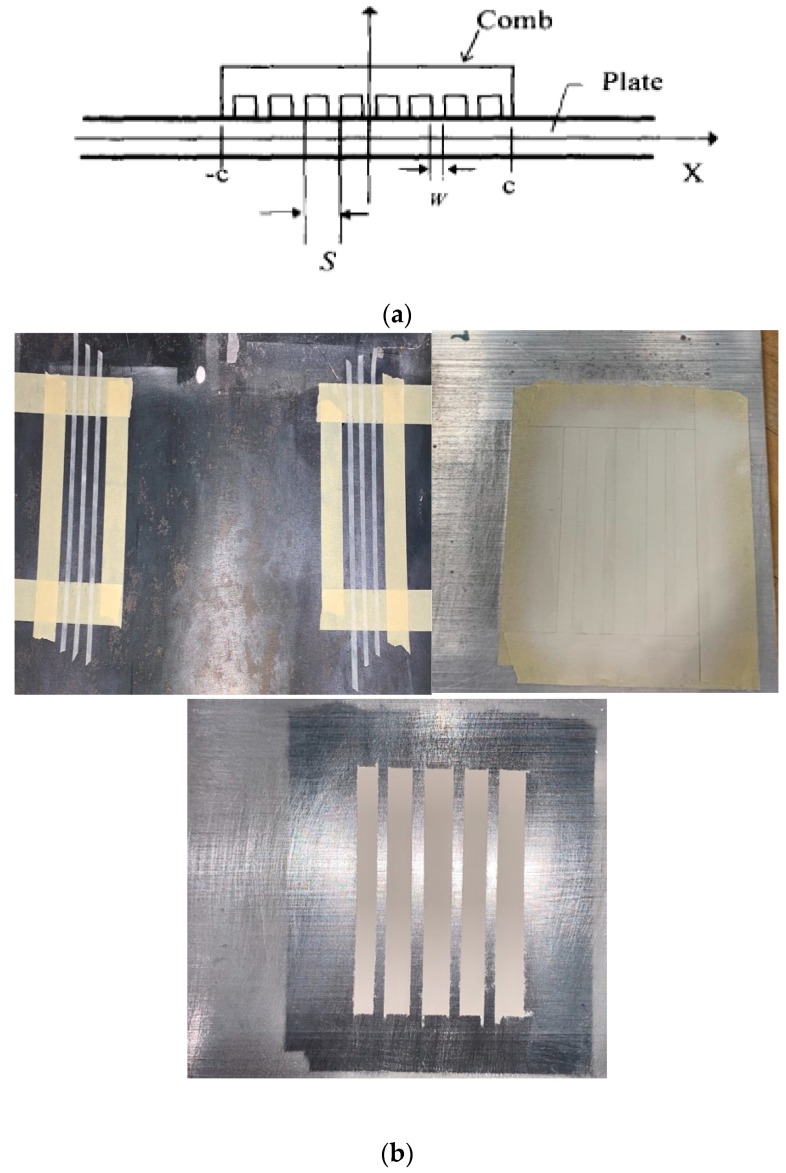
(**a**) S is the element width, W is the element gap, W + S is the wavelength of the traveling wave [7]; (**b**) Schematic of the experiment setup for generation of Lamb waves in a 3.2 mm thick 6061 aluminum plate [7].

To demonstrate their ability to perform as a guided-wave sensor, the primary Lamb wave modes (A0, S0, A1, S1) were generated using a comb transducer arrangement. Furthermore, 6061 aluminum plates of several different thicknesses ranging from 2 to 4 mm were chosen as the waveguides. Sets of comb transducers were then applied onto the plate a certain distance apart in a through-transmission setup as shown in Figure 16a,b.

**Figure 17:**
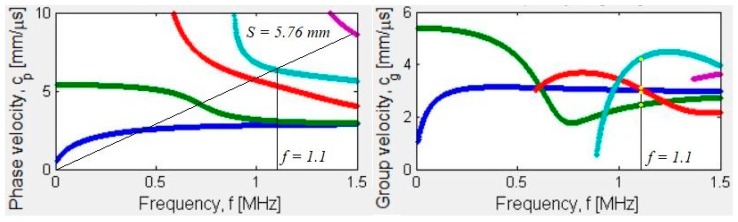
Dispersion curves for a 3.2 mm thick aluminum 6061 plate showing the activation line on the phase velocity and corresponding group velocities at that frequency [82].

**Figure 18:**
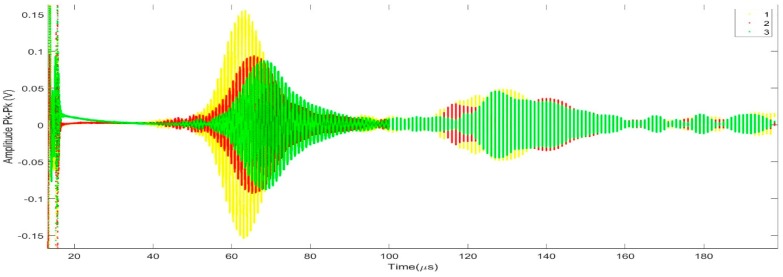
A-scan for one of the tested plates showing the superimposed guided-wave modes from three different elements [7].

The number of actuating and receiving elements were altered to give rise to various configurations for better transducer characterization. Calculations solving the thin-plate Lamb wave transcendental equations were performed for the comb elements to be spaced by the same length as the wavelength of the preferential excited mode [79]. See Figure 17 for graphs of the corresponding dispersion curves. A tone-burst of 15 cycles was introduced in the actuator set of the transducers and the A-scan was plotted. According to the excitation parameters and the comb spacing, the S1 mode should be the first to be received as shown in Figure 18. The readings were then recorded through various receiving elements and calculation were performed to compare the experimental group velocity for S1 mode with its theoretical value at that specific frequency.

**Figure 19:**
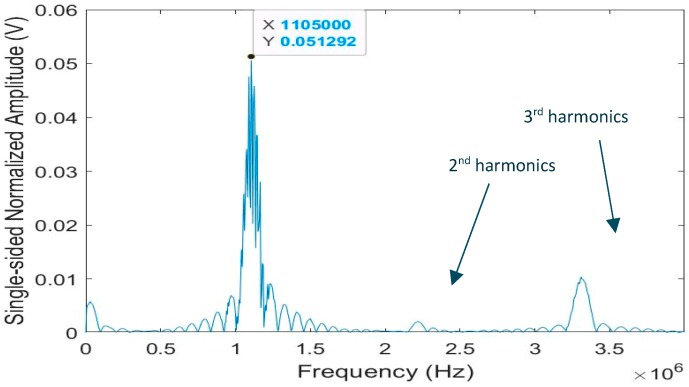
Frequency spectrum for one of the guided modes. The presence of harmonics is also indicated [7].

Figure 19 presents the frequency spectrum for one of the received waveforms showing both the second and third harmonics. The third harmonic appears relatively strong and useful for further studies. The other A-scan waveforms display similar frequency spectra.

Signal-to-noise ratio (SNR)was calculated by taking the ratio of the root-mean-square (rms) value of the amplitude (peak-to-peak) within the mode window to the rms value of the noise window in decibel units given by this formula.

$$SNR=20*{log}_{10}\left(\frac{V_{pk-to-pk}}{V_{noise}}\right)$$

The rms value is calculated by multiplying 1/(2√2) to the peak-to-peak voltage. The signal-to-noise ratio along with the signal strength for the three films is shown in Table 5.

The inorganic films are easy to produce and extremely inexpensive, as the films use very small quantities. A batch of 5 g powder with another 5 g of the solvents is enough to coat at least two of the stainless cubes used for the pulse-echo measurements. For spraying films on bigger areas, the slurry quantity would also have to be increased, but compared to the cost of commercial sensors these transducers are affordable. The respective SNR of 20.72, 14.93, and 19.93 dB for the Bi_4_Ti_3_O_12_, LiNbO_3_, and organic Bi_4_Ti_3_O_12_ films is comparable to commercial transducers. According to the study done by Kobayashi, the films on the planar surfaces yielded an SNR of 16 dB and a center frequency of 3.6 MHz, where the Bi_4_Ti_3_O_12_ was doped with PZT. Here, without a strong piezoelectric material such as PZT, the signal strength is quite strong. The center frequencies for the Bi_4_Ti_3_O_12_, LiNbO_3_, and organic Bi_4_Ti_3_O_12_ films were 1.4, 1.22, and 1.42 MHz, respectively. With the premise of a working transducer, the films were then subjected to temperature testing. From the capacitance, other parameters to characterize the films could be calculated such as the dielectric constant, dielectric loss, amount of charge in the film, and even piezoelectric constant after some analysis. The higher the dielectric constant, more charge could be held by it and used as electric potential. Poling these films requires patience. Through some further study, it was found that an aluminum oxide protective layer on the top of the piezo film was useful for serving as an electrical blanket, which prevented the charges from jumping and short circuiting the film. With the alumina layer, the films could be poled at voltages only a few hundred volts below the coercive field.

## 5. Conclusions

This Invited Special Issue contribution addresses the current state-of-the-art and offers a practical guide to ultrasonic transducers for harsh environments including temperatures above 2120 °F (1000 °C) and neutron flux above 10^13^ n/cm^2^.

In field applications currently used for health monitoring and nondestructive testing, ultrasonic transducers primarily employ PZT5-H as the piezoelectric element for ultrasound transmission and detection. This material has a Curie–Weiss temperature which limits its use to about 210 °C. Some industrial applications require much higher temperatures, i.e., 1000–1200 °C and possible nuclear radiation up to 10^20^ n/cm^2^ when performance is required in a reactor environment.

The goal of this paper is the survey and review of piezoelectric elements for use in harsh environments for the ultimate purpose for Structural Health Monitoring (SHM), Non-destructive Evaluation (NDE) and material characterization (NDMC). The survey comprised the following categories: 1. High temperature applications with single crystals, thick film ceramics, and composite ceramics, 2. Radiation tolerant materials, and 3. Spray-on transducers for harsh environment applications. In each category the known characteristics are listed, and examples are given of performance in harsh environments.

In summary we have presented a survey of piezoelectric materials capable of operation at higher temperatures and possible nuclear radiation. This survey tries to both gather information and summarize it. The findings indicate that PZT/Bi_4_Ti_3_O_12_ and Bi_4_Ti_3_O_12_/LiNbO_3_ composite transducers functioned in pulse-echo mode until 675 and 1000 °C, respectively. Recent interest in the radiation endurance of piezoelectric ultrasonic transducers has stimulated a search for appropriate materials. Some applications may be found in materials research reactors where ultrasonic NDE can be used for in situ analysis of radiation effects on novel radiation-hard materials currently being developed. This paper presents a survey of piezoelectric materials for possible harsh-environment applications. Moreover, our experiments in a nuclear reactor for one of the materials, AlN, demonstrated an example of possible resistance to radiation. Unfortunately, AlN is not a very efficient producer of ultrasonic waves. Therefore, one of the future goals is to come up with transducers with higher efficiency that are tolerant of radiation. We also showed that some of the high-temperature transducers could be mounted on a target without requiring coupling material. Guided-wave send-and-receive was demonstrated on planar and pipe structures for possible field deployable applications. Interesting and possibly relevant research applications with brush-on transducers are going on at the University of Montpellier for fission gas characterization [83,84].

## Figures and Tables

**Table 1:** Some well-known piezoelectrics [1,2,3,4,5,6,7,8,9].

Piezoelectric Material	Curie–Weiss Temperature (°C)
PZT-5H	210
Keramos lead metaniobate	400
Bismuth titanate	685
Lithium niobate	1000

**Table 2:** High-temperature piezoelectric ceramics.

Piezoelectric Material	Curie Temperature (°C)
Praseodymium titanate	>1550 [11,12]
Lanthanum titanate	1461 [13,16]
Neodymium titanate	1482 [13,17]
Strontium niobate	1327 [14]
Calcium niobate	>1525 [15]

**Table 3:** Piezoelectric materials [33].

Material	Transition Temperature °C	Transition Type	Structure
AlN	2826	Melt	Wurtzite [6]
Bi_3_TiNbO_9_	909	Curie	Perovskite layered [32]
LiNbO_3_	~1200	Curie	Perovskite [21]
Sr_2_Nb_2_O_73_	1342	Curie	Perovskite layered [32]
La_2_Ti_2_O_7_	1500	Curie	Perovskite layered [32]
GaPO_4_	970	α-β	SiO_2_ homeotype [32]
ReCa_4_(BO_3_)_3_, *Re as Rare Earth element*	>1500	Melt	Oxyborate homeotype [32]
ZnO	1975	Melt	Wurtzite [33]

**Table 4:** MIT research reactor environment.

The Massachusetts Institute of Technology Reactor is characterized by the following features:
Total flux = 1.89 × 10^14^ n/cm^2^
Thermal flux (<0.4 eV) = 2.12 × 10^13^ n/cm^2^
Epi-thermal flux (0.4 eV–0.1 MeV) = 8.03 × 10^13^ n/cm^2^
Fast flux 1 (>0.1 MeV) = 8.78 × 10^13^ n/cm^2^
Fast flux 2 (>1.0 MeV) = 4.05 × 10^13^ n/cm^2^
Gamma dose rate: 1 × 10^9^ r/h
Temperature: 400–500 °C

**Table 5:** Signal-to-noise calculations for the inorganic and organic films.

Film	Bi_4_Ti_3_O_12_	LiNbO_3_	Organic Bi_4_Ti_3_O_12_
**Signal window (μs)**	5.071–5.739	5.071–5.554	5.030–5.635
**Noise window (μs)**	5.756–7.510	5.615–7.490	5.675–7.450
**Signal strength pk-pk (V)**	2.895	0.868	2.815
**Noise strength pk-pk (V)**	0.267	0.156	0.284
**Signal rms (V)**	1.024	0.307	0.996
**Noise rms (V)**	0.094	0.055	0.100
**Signal strength (dB)**	9.321	−1.232	8.990
**Noise strength (dB)**	−11.484	−16.157	−10.939
**Signal-to-noise ratio (dB)**	20.716	14.926	19.929
